# Comparative Study of *Phellodendron amurense* Rupr. Components in Alleviating Diabetic Kidney Disease via the PI3K/AKT/GSK-3β/Nrf2 Pathway

**DOI:** 10.3390/ph19060965

**Published:** 2026-06-22

**Authors:** Mei Mei, Huawei Sun, Kai Zhang, Feng Zhang, Shiqing Sun, Enbin Yu, Yu Zhang

**Affiliations:** 1 Key Laboratory of Microecology-Immune Regulatory Network and Related Diseases School of Basic Medicine, Jiamusi University, Jiamusi 154007, China; dminn2017@163.com (M.M.); zhangkai930222@163.com (K.Z.); zhangfeng3618@163.com (F.Z.); ssq19971030@163.com (S.S.); 18332602785@163.com (E.Y.); 2 The First Affiliated Hospital, Jiamusi University, Jiamusi 154002, China; shwmm@21cn.com; 3 College of Pharmacy, Jiamusi University, Jiamusi 154007, China

**Keywords:** *Phellodendron amurense* Rupr., diabetic kidney disease, PI3K/AKT/GSK-3β/Nrf2 pathway

## Abstract

**Background:** To investigate the protective effects of *Phellodendron amurense* Rupr. polysaccharides (PAP), alkaloids, and flavonoids in alleviating diabetic kidney disease (DKD) and to elucidate the role of the PI3K/AKT/GSK-3β/Nrf2 signaling pathway. **Methods:** Active components were extracted and quantified. In vitro, high-glucose (HG)-induced human kidney-2 (HK-2) cells were used to screen the optimal fraction via CCK-8, reactive oxygen species (ROS), TdT-mediated dUTP Nick-End Labeling (TUNEL), and Western Blot (WB) assays. In vivo, a DKD rat model was established using 2% Streptozotocin (STZ) and a high-fat with high-sugar diet. Rats were treated with PAP and LY294002. Renal damage and signaling pathway proteins were evaluated using histological staining and WB. **Results:** Among the tested components, PAP conferred the most pronounced cytoprotection against HG-induced injury in HK-2 cells. PAP significantly reduced glomerular damage, collagen deposition, and glycogen accumulation in the kidneys of DKD rats. Mechanistically, PAP activated the PI3K/AKT/GSK-3β/Nrf2 pathway, upregulating HO-1 and NQO1, while inhibiting the TGF-β1/Smad2 pathway and Bcl-2/Bax-mediated apoptosis. These protective effects were significantly attenuated by LY294002. **Conclusions:** Among the tested fractions under the present experimental conditions, PAP exhibited the most pronounced protective activity. These protective effects were partially mediated through the PI3K/AKT/GSK-3β/Nrf2 pathway, which enhanced antioxidant capacity while reducing fibrosis and apoptosis.

## 1. Introduction

Diabetes is a severe, chronic, and progressive metabolic disease. According to the latest epidemiological survey by the International Diabetes Federation (IDF), as of 2020, the number of people living with diabetes globally was nearly 460 million. The prevalence rate is projected to rise from 9.3% in 2019 to 10.2% by 2030, and the number of people with diabetes is expected to reach 693 million by 2045, with approximately half of those affected remaining undiagnosed [1]. Diabetic kidney disease (DKD) is a multifaceted disease involving various mechanisms. Currently, the mainstream view suggests that the pathogenesis of DKD is primarily related to inflammatory responses, oxidative stress, and apoptosis [2].

Oxidative stress is a way for the body to respond to adverse external stimuli, where a dynamic equilibrium is maintained between the oxidative and antioxidant systems under physiological conditions. During cellular metabolism, free radicals, also known as reactive oxygen species (ROS), are continuously formed. ROS serve as regulators of intracellular signaling pathways that control cell growth, differentiation, and many other processes [3,4]. ROS levels are crucial for maintaining various cellular and tissue functions as well as the progression of DKD. Simultaneously, ROS could activate TGF-β [5]. TGF-β functions as a primary signaling molecule in the progression of DKD. It drives the apoptosis of renal tubular epithelial cells and contributes to their transdifferentiation by utilizing the TGF-β-Smad2/3 signaling pathway. Consequently, these processes facilitate the accumulation of renal extracellular matrix (ECM), which eventually culminates in renal fibrosis associated with DKD [6]. The activation of the TGF-β/Smad signaling cascade has the potential to stimulate the synthesis of ECM and elevate cellular α-SMA levels. This triggers an overaccumulation of the extracellular matrix, ultimately disrupting regular kidney architecture and impairing its physiological function [7]. Moreover, GSK-3β is able to phosphorylate Nrf2, facilitating its association with Keap1 and triggering its subsequent ubiquitination and degradation, which effectively suppresses Nrf2 activity [8]. Furthermore, the active form of GSK-3β modulates the Bax/Bcl-2 proportion, altering the permeability of the mitochondrial membrane. This process triggers the opening of mitochondrial permeability transition pores and drives the efflux of Cyto C from the mitochondria, thereby playing a definitive role in mediating cell apoptosis. Consequently, suppressing the generation of ROS to relieve oxidative stress, renal fibrosis, and apoptosis serves as a crucial strategy for ameliorating DKD.

*Phellodendron amurense* Rupr., as a traditional medicine for clearing heat and drying dampness, contains various active ingredients such as alkaloids, polysaccharides, and flavonoids, and possesses significant anti-inflammatory, antioxidant, and metabolic regulatory activities. Although the role of alkaloids such as berberine in the treatment of diabetes has received extensive attention, the pharmacodynamic material basis of *Phellodendron amurense* Rupr. as a complex compound in preventing and treating DKD remains incompletely clarified. In particular, there is a lack of systematic research regarding the specific pharmacodynamic effects of non-alkaloid components, such as *Phellodendron amurense* Rupr. polysaccharides (PAP), in DKD and whether they act by regulating the Nrf2 antioxidant pathway. This project utilizes a high-glucose (HG) induced human kidney-2 (HK-2) cell model in vitro, and a DKD rat model induced by 2% Streptozotocin (STZ) solution and a high-fat and high-sugar diet. Based on different extraction and separation methods, we compare the pharmacodynamic effects of different effective fractions of *Phellodendron amurense* Rupr. in alleviating DKD and select the optimal effective fraction to study the mechanism of action underlying DKD alleviation through in vivo models.

## 2. Results

### 2.1. Screening of Active Fractions in HG-Induced HK-2 Cells

#### 2.1.1. Cell Viability and Optimal Dose Screening

**Figure 1:**
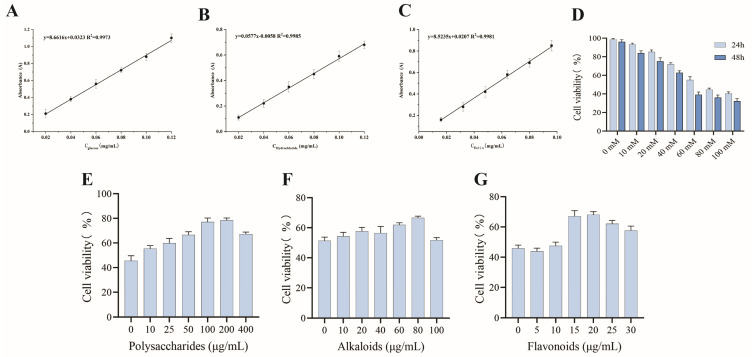
Quantitative standard curves of active components and screening of the HG induction concentration and the optimal administration concentrations of active fractions in HK-2 cells. (**A**) Standard curves of glucose; (**B**) Standard curves of hydrochloride; (**C**) Standard curves of rutin; (**D**) Cell viability of HK-2 cells treated with varying concentrations of glucose (0–100 mM) for 24 h and 48 h to determine the optimal HG-induced injury model; (**E**) Cell viability of HG-induced HK-2 cells treated with different concentrations of polysaccharides; (**F**) Cell viability of HG-induced HK-2 cells treated with different concentrations of alkaloids; (**G**) Cell viability of HG-induced HK-2 cells treated with different concentrations of flavonoids.

After extraction, separation, and preliminary purification, polysaccharides, alkaloids, and flavonoids were obtained from *Phellodendron amurense* Rupr. Based on the quantitative standard curves (Figure 1A–C), the extraction yield and content of total polysaccharides were 6.32 ± 0.42% and 73.26 ± 1.55%, respectively. The extraction yield of total alkaloids was 2.35 ± 0.24%, with a content of 60.15 ± 2.03%. Similarly, the extraction yield of total flavonoids was 3.27 ± 0.27%, with a content of 63.32 ± 1.64%. Subsequently, a HG-induced HK-2 cell model was utilized to select the administration concentrations of the effective fractions and to determine the working concentrations for subsequent experiments. High glucose at 60 mM significantly inhibited the viability of human kidney 2 (HK-2) cells at 24 h; therefore, 60 mM was selected as the optimal induction concentration to establish the injury model (Figure 1D). To determine the effective working concentrations, the viabilities of HG-induced HK-2 cells treated with varying concentrations of polysaccharides (Figure 1E), alkaloids (Figure 1F), and flavonoids (Figure 1G) were evaluated. The results demonstrated that the optimal administration concentrations were 50 μg/mL for total polysaccharides, 20 μg/mL for total alkaloids, and 15 μg/mL for total flavonoids. These specific concentrations were employed for all subsequent experiments.

#### 2.1.2. Effects of Active Fractions on Cell Morphology, ROS, and Apoptosis

**Figure 2:**
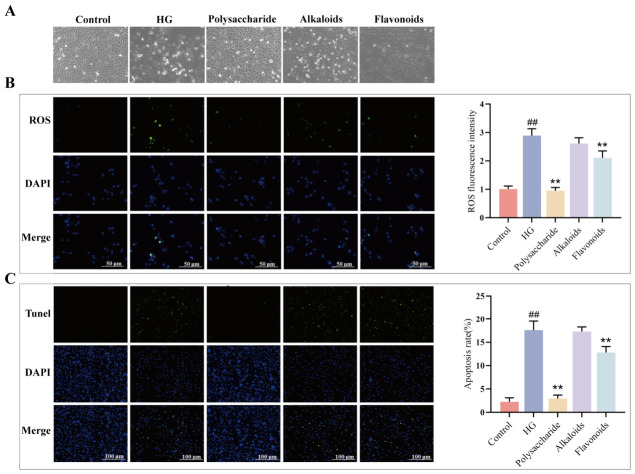
The effects of active fractions from *Phellodendron amurense* Rupr. on HG-induced injury in HK-2 cells. (*n* = 3) (**A**) Representative images of cell morphological structures in different treatment groups; (**B**) Representative fluorescence images of intracellular ROS production and the corresponding quantitative histogram of ROS fluorescence intensity (**C**) Representative fluorescence images of TUNEL staining and the corresponding quantitative histogram of the apoptosis rate (^##^ *p* < 0.01 vs. Control group; ** *p* < 0.01 vs. HG group).

As shown in Figure 2A, cells in the Control group exhibited a cobblestone-like paving pattern with tight junctions. Compared with the Control group, HG-induced HK-2 cells transformed into a long spindle shape with enlarged intercellular spaces. In contrast to the HG group, the polysaccharides and flavonoids from *Phellodendron amurense* Rupr. could alleviate the abnormalities in HK-2 cells induced by high glucose, while the alkaloids showed only a mild and negligible improvement. To investigate the effects of the effective fractions of *Phellodendron amurense* Rupr. on the antioxidant levels of HG-induced HK-2 cells, the intracellular ROS fluorescence intensity was detected. As shown in Figure 2B, the ROS fluorescence intensity in HK-2 cells significantly increased under HG induction (*p* < 0.01). Compared with the HG group, both polysaccharides and flavonoids significantly reduced the ROS levels in HK-2 cells (*p* < 0.01), while alkaloids reduced the ROS levels but without statistical significance. To examine the effects of the effective fractions on the anti-apoptotic levels of high-glucose-induced HK-2 cells, the intracellular TUNEL fluorescence intensity was measured. As shown in Figure 2C, the TUNEL fluorescence intensity in HK-2 cells significantly increased under high-glucose induction (*p* < 0.01). Compared with the HG group, both polysaccharides and flavonoids significantly reduced the TUNEL positive levels in HK-2 cells (*p* < 0.01), whereas alkaloids showed a reduction in levels without statistical significance. In summary, polysaccharides and flavonoids could mitigate high-glucose-induced HK-2 cell injury and enhance antioxidant and anti-apoptotic levels, with PAP exhibiting the better pronounced effect than flavonoids.

#### 2.1.3. WB Analysis of Cells Treated with Active Fractions

**Figure 3:**
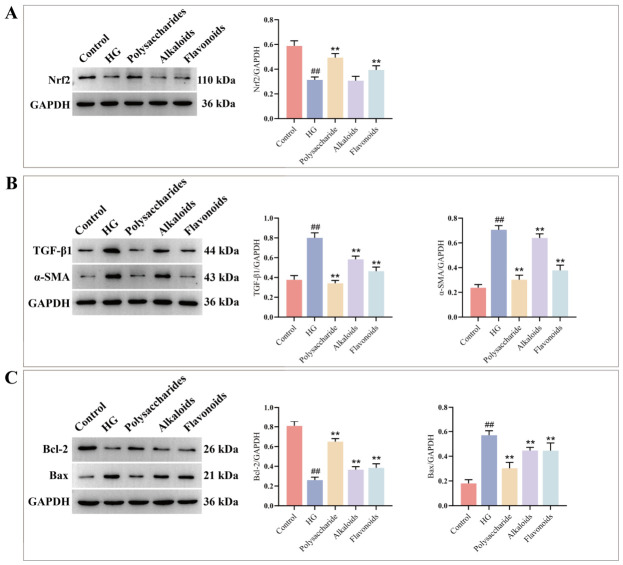
The effects of active fractions from *Phellodendron amurense* Rupr. on the protein expressions of Nrf2, TGF-β1, α-SMA, Bcl-2 and Bax in HG-induced HK-2 cells. (*n* = 3) (**A**) WB bands of Nrf2; (**B**) WB bands of TGF-β1 and α-SMA; (**C**) WB bands of Bcl-2 and Bax. (^##^ *p* < 0.01 vs. Control group; ** *p* < 0.01 vs. HG group). Figure S1 contains the original uncropped Western blot images for Figure 3.

To further investigate the effects of active fractions from *Phellodendron amurense* Rupr. against HG-induced injury in HK-2 cells, the expression levels of proteins associated with oxidative stress, fibrosis, and apoptosis were evaluated via Western blotting. HG exposure significantly downregulated the expression of the antioxidant protein Nrf2 (Figure 3A) and the anti-apoptotic protein Bcl-2 (Figure 3C), while markedly upregulating the profibrotic proteins TGF-β1 and α-SMA (Figure 3B) as well as the pro-apoptotic protein Bax (Figure 3C) compared to the Control group. Following intervention, all three active fractions significantly ameliorated HG-induced fibrotic and apoptotic responses by downregulating TGF-β1, α-SMA, and Bax, and robustly restoring Bcl-2 expression (*p* < 0.01). Regarding antioxidant capacity, both the polysaccharides and flavonoids groups significantly increased Nrf2 expression compared to the HG group (*p* < 0.01), whereas the alkaloids group exhibited no significant impact on Nrf2 levels.

### 2.2. PAP Improves Renal Injury in DKD Rats via the PI3K/AKT/GSK-3β/Nrf2 Signaling Pathway

#### 2.2.1. Histological Evaluation of Renal Tissues

**Figure 4:**
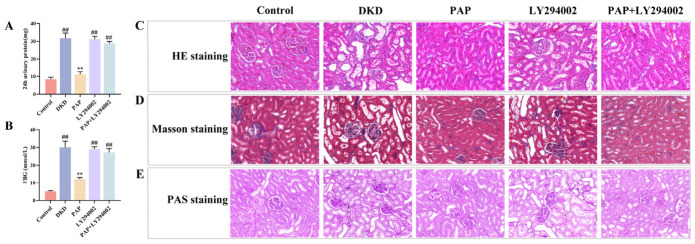
The effects of PAP and LY294002 on renal function and histopathological alterations in DKD rats. (**A**) 24h urinary protein levels in DKD rats; (**B**) FBG levels in DKD rats; (**C**) Representative images of HE staining (200×); (**D**) Representative images of Masson staining (200×); (**E**) Representative images of PAS staining (200×). (^##^ *p* < 0.01 vs. Control group; ** *p* < 0.01 vs. DKD group).

The levels of 24 h urinary protein (Figure 4A) and fasting blood glucose (FBG) (Figure 4B) were significantly elevated in the DKD group compared to the Control group (*p* < 0.01). Although PAP intervention markedly reduced these physiological parameters (*p* < 0.01), this therapeutic effect was substantially abolished by the addition of the PI3K inhibitor LY294002, with the PAP + LY294002 co-treatment group showing persistently high levels.

HE staining (Figure 4C) revealed that the rats in the Control group possessed intact glomerular structures with no abnormal changes in the matrix or mesangium, and the renal tubules were structurally complete. In the DKD group, significant necrosis was observed in the kidney tissues, accompanied by glomerular atrophy, extensive degeneration of renal tubular epithelial cells, and a large number of inflammatory cells. In the PAP group, the degree of glomerular basement membrane thickening was significantly improved, and the proliferation of the glomerular mesangial matrix was markedly reduced. Treatment with LY294002 alone increased renal tissue necrosis, glomerular atrophy, and extensive degeneration of renal tubular epithelial cells. Crucially, the protective effect of PAP was slightly reversed in the PAP + LY294002 group, which exhibited severe pathological damage similar to the DKD group. Masson staining (Figure 4D) showed trace amounts of collagen in the glomerular basement membrane and renal tubulointerstitium of the Control group. In contrast, abundant collagen fibers were observed in the glomeruli and renal tubules of the DKD group. The PAP group exhibited fewer collagen fibers in the glomeruli and renal tublointerstitium compared to the DKD group. While collagen fibers increased in the renal tissues with LY294002 treatment alone, the PAP + LY294002 group showed improvement. PAS staining (Figure 4E) demonstrated that, compared with the Control group, the PAS-positive staining in the glomerular mesangial area of the DKD group was significantly increased. Compared with the DKD group, the positive expression in the PAP group was markedly reduced, indicating that the accumulation of PAS-positive material in the PAP group was significantly improved and that PAP can notably reduce the levels of PAS-positive in renal tissues. While PAS-positive expression in the glomerular mesangial area was significantly higher with LY294002 alone, there was a improvement following the administration of PAP + LY294002.

#### 2.2.2. Immunofluorescence Analysis of Nrf2 and p-GSK-3β

**Figure 5:**
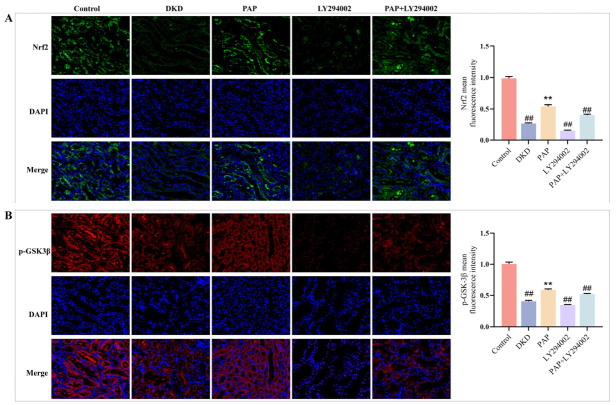
The effects of PAP and LY294002 on the protein expressions of Nrf2 and p-GSK-3β in the DKD rats. (n = 3) (**A**) Representative immunofluorescence images and quantitative analysis of Nrf2 mean fluorescence intensity; (**B**) Representative immunofluorescence images and quantitative analysis of p-GSK-3β mean fluorescence intensity. (^##^ *p* < 0.01 vs. Control group; ** *p* < 0.01 vs. DKD group).

To investigate the effects of PAP on the expression of Nrf2 and p-GSK3β in the DKD model, and to verify whether this process is mediated by the PI3K pathway, we performed immunofluorescence staining. Compared with the Control group, the protein expression levels of Nrf2 (Figure 5A) and p-GSK3β (Figure 5B) were significantly decreased in the DKD group. Following PAP intervention, the fluorescence signals of both proteins increased significantly (*p* < 0.01). However, co-treatment with the PI3K inhibitor LY294002 attenuated the up regulatory effect of PAP on Nrf2 and p-GSK3β expression. Notably, this restorative effect of PAP was significantly reversed in the PAP + LY294002 co-treatment group (*p* < 0.01). Taken together, these results indicate that PAP upregulates the expression of Nrf2 and p-GSK3β in DKD, and this modulatory effect is dependent on the PI3K signaling pathway.

#### 2.2.3. Regulation of PI3K/AKT/GSK-3β, TGF-β1/Smad2, and Apoptosis Pathways by PAP

**Figure 6:**
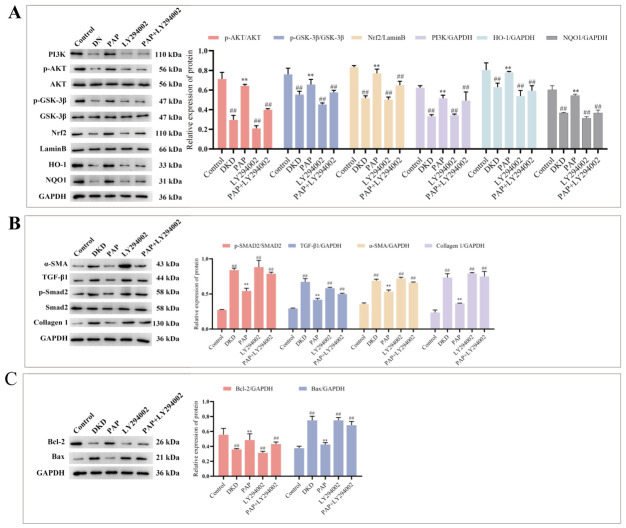
The effects of PAP and LY294002 on the protein expressions of the PI3K/AKT/GSK-3β/Nrf2 pathway, TGF-β1/Smad pathway, and apoptosis-associated proteins in the DKD rats. (*n* = 3) (**A**) Representative Western blot images and quantitative analysis of p-AKT/AKT, p-GSK-3β/GSK-3β, Nrf2/LaminB, PI3K/GAPDH, HO-1/GAPDH, and NQO1/GAPDH; (**B**) Representative Western blot images and quantitative analysis of p-Smad2/Smad2, TGF-β1/GAPDH, α-SMA/GAPDH, and Collagen 1/GAPDH; (**C**) Representative Western blot images and quantitative analysis of Bcl-2/GAPDH and Bax/GAPDH. (^##^ *p* < 0.01 vs. Control group; ** *p* < 0.01 vs. DKD group). Figure S1 contains the original uncropped Western blot images for Figure 6.

To investigate the underlying molecular mechanisms of PAP in DKD rats, protein expression levels were evaluated using Western blot analysis. The induction of DKD significantly suppressed the PI3K/AKT/GSK-3β oxidative stress pathway, evidenced by the marked downregulation of PI3K, p-AKT/AKT, and p-GSK-3β/GSK-3β, along with downstream antioxidant factors that nuclear Nrf2, HO-1, and NQO1 compared to the Control group (*p* < 0.01) (Figure 6A). Treatment with PAP significantly restored these protein expressions (*p* < 0.01), whereas co-treatment with the PI3K inhibitor LY294002 significantly attenuated this PAP-induced upregulation (*p* < 0.01). Regarding the TGF-β1/Smad2 fibrosis pathway (Figure 6B), the expression levels of pro-fibrotic markers, including α-SMA, TGF-β1, p-Smad2/Smad2, and Collagen 1, were notably elevated in the DKD group (*p* < 0.01); PAP administration effectively inhibited this overexpression (*p* < 0.01), but this anti-fibrotic effect was reversed by the addition of LY294002 (*p* < 0.01). Furthermore, to evaluate cell apoptosis (Figure 6C), results showed that the DKD group exhibited a significant upregulation of Bax and downregulation of Bcl-2 (*p* < 0.01). PAP treatment effectively mitigated this apoptotic state (*p* < 0.01), while LY294002 significantly diminished the protective anti-apoptotic effect of PAP (*p* < 0.01). Collectively, these results comprehensively confirm that the anti-oxidative, anti-fibrotic, and anti-apoptotic protective effects of PAP in DKD rats are partially mediated through the activation of the PI3K/AKT signaling pathway.

## 3. Discussion

DKD is a complex and progressive microvascular complication whose pathogenesis is intimately associated with pathophysiological processes such as oxidative stress, renal fibrosis, and apoptosis [9]. The search for natural medicines capable of multi-target intervention and delaying disease progression has thus become an important research direction. In recent years, increasing attention has been paid to the value of *Phellodendron amurense* Rupr. in specific diseases, including its antioxidant [10], hypoglycemic [11], and nephroprotective effects [12].

In this study, we systematically evaluated and compared the protective effects of the polysaccharide, alkaloid, and flavonoid fractions of *Phellodendron amurense* Rupr. against HG-induced injury in HK-2 cells at the in vitro level. Although the alkaloid and flavonoids fractions downregulated fibrotic and apoptotic proteins to a certain extent, PAP exhibited the better comprehensive and superior anti-injury efficacy than alkaloid, and flavonoid. Moreover, in vitro results showed that while the alkaloid fraction could reduce the expression of the intracellular fibrotic markers TGF-β1 and α-SMA to some degree, it did not display statistically significant activity in ameliorating high glucose-induced ROS accumulation or in suppressing apoptosis. Currently, more than 79 alkaloid components have been isolated or identified from *Phellodendron amurense* Rupr., alkaloids are the most widespread and also the earliest reported components in this species [13]. Common alkaloids include berberine, palmatine, jatrorrhizine, and others [14,15]. Moreover, berberine-type alkaloids have been found to possess good antioxidant, anti-inflammatory, anti-proliferative, and other activities [16,17].

Researchers have identified four flavonoids and their glycosides by UPLC-Q-TOF-MS [18]. It has also been found that flavonoids from *Phellodendron amurense* Rupr. exhibit multiple activities, including antioxidant, anticancer, and hypoglycemic effects [19,20]. However, it must be noted that because the optimal effective concentrations and natural abundances differ among the various extract fractions, the conclusion that PAP is the most effective fraction currently represents a preliminary judgment based on the present experimental conditions. In the future, more rigorous equivalence comparisons on an equimolar or equal crude drug basis, combined with the actual extraction yields of each fraction, are required to definitively identify the pharmacologically dominant fraction. In the in vitro HK-2 cell model induced by 60 mM high glucose, PAP still demonstrated an extremely potent intracellular ROS scavenging capacity and significantly reduced the apoptotic rate of TUNEL-positive cells. These results indicate that PAP possesses an intrinsic, locally direct protective mechanism on renal tubular epithelial cells that is independent of systemic blood glucose reduction. It should be acknowledged that 60 mM glucose is mainly used to establish a short-term acute glucotoxicity model, which differs to some extent from the chronic pathological state of diabetic patients enduring long-term hyperglycemia. Additionally, the absence of isotonic control in experimental design means that the contribution of hyperosmotic stress-induced damage to HK-2 cells cannot be completely excluded.

Previous research has revealed that PAP has a molecular weight of approximately 1.98 × 10^5^ Da and is primarily composed of rhamnose, galacturonic acid, galactose, and D-xylose. Extensive recent studies have shown that plant-derived macromolecular polysaccharides rich in such monosaccharides generally possess strong free radical-scavenging abilities and the potential to target improvements in type 2 diabetes metabolism [21]. However, unlike previously reported nephroprotective studies on Astragalus polysaccharides or Angelica sinensis polysaccharides that mainly focus on antioxidant or immunomodulatory effects, this study observed that PAP simultaneously and significantly inhibits both the TGF-β1/Smad2-mediated fibrotic pathway and the Bcl-2/Bax-mediated apoptotic pathway in a DKD model. This dual regulatory feature of antioxidant and anti-fibrotic activity is relatively uncommon among polysaccharides, providing new evidence for natural polysaccharides in the treatment of complex metabolic diseases. Astragalus Mongholicus Polysaccharides can alleviate renal injury in high-sugar and high-fat diet coupled with STZ-induced diabetic rats by activating the Nrf2/Keap1 signaling pathway and modulating the gut microbiota, and are primarily composed of mannose, ribose, arabinose, glucose, galactose, xylose, glucuronic acid, and galacturonic acid [22,23]. Polysaccharides extracted from okra (*Abelmoschus esculentus* (L.) Moench) exhibit significant hypoglycemic effects, with the underlying mechanism involving the PI3K/AKT pathway and Nrf2 nuclear translocation; these polysaccharides are mainly composed of mannose, rhamnose, glucuronic acid, galacturonic acid, galactose, and arabinose [24]. However, the molecular weights of Astragalus polysaccharides and A. esculentus polysaccharides have not been reported. Furthermore, degraded Noni juice polysaccharides (Mw 191.8 kDa) can ameliorate glucose metabolism disorders in HepG2 cells via the PI3K/AKT-Nrf2-GSK3β signaling pathway [25]. In the in vivo DKD rat model, the experimental animals exhibited typical clinical signs such as persistent hyperglycemia, pathological structural damage to the kidneys, and increased urinary protein excretion. Long-term PAP intervention significantly improved renal function and renal histomorphology. Notably, the highest dose used in this study, 800 mg/kg, was converted to a human equivalent dose of approximately 129.7 mg/kg based on body surface area (equivalent to roughly 7.8 g/day for a 60 kg adult). This dose falls within the feasible oral administration range for natural polysaccharide preparations, and the fact that no obvious toxic reactions were observed during the 8-week treatment period indicates that PAP is safe at this dosage.

The PI3K/AKT cascade is fundamental in governing vital cellular activities, including glucose metabolism, programmed cell death, differentiation, and proliferation. By utilizing a DKD rat model induced by 2% STZ, which presents characteristic clinical and pathological traits of the disease, our histological assessments clearly illustrated that PAP substantially mitigated glomerular damage. It also restricted abnormal glycogen accumulation and excessive collagen deposition, thereby preserving overall renal architecture. Molecular evaluations via Western blot revealed that PAP administration effectively stimulated the PI3K/AKT/GSK-3β/Nrf2 axis within the renal tissues of the DKD model, upregulating the associated proteins while profoundly suppressing both the TGF-β/Smad cascade and Bcl-2/Bax-driven apoptosis. Furthermore, PAP elevated the levels of proteins typically suppressed by LY294002-induced AKT inactivation. Together, these in vivo findings suggest that PAP combats DKD-related renal impairment by orchestrating the AKT/GSK-3β/Nrf2 regulatory network. The progression of DKD is heavily driven by oxidative stress. The overproduction of reactive ROS triggers multiple cascades—including the hexosamine, JAK-STAT, MAPK, and PKC pathways—which ultimately promote tubulointerstitial fibrosis, inflammation, cellular senescence, apoptosis, and extracellular matrix expansion in the glomeruli, thereby accelerating the deterioration toward end-stage renal failure [26,27]. Both normal physiological functions and pathological responses are modulated by the PI3K/AKT axis [28]. Existing literature highlights the role of PI3K in regulating Nrf2 activation and its subsequent nuclear import [29]. Specifically, PI3K facilitates the dissociation of Nrf2 from Keap1 by altering actin dynamics and promoting depolymerization, thereby accelerating Nrf2 translocation. Because AKT serves as a primary downstream effector of PI3K, its phosphorylation status acts as a reliable indicator of PI3K activation. Functioning as a primary cellular safeguard against oxidative damage, Nrf2 drives the transcription of antioxidant genes to clear ROS and preserve redox balance [30] Our findings demonstrate a progressive reduction in p-AKT and Nrf2 protein levels as the disease advances. We hypothesize that during the initial phases of DKD, the body exhausts its Nrf2 reserves to upregulate downstream antioxidant defenses in an effort to mitigate ongoing injury. By bolstering this antioxidant capacity, PAP successfully suppressed oxidative injury and mitigated renal fibrosis. Mitochondrial dynamics are heavily regulated by GSK-3β, a known downstream target of AKT [31]. Once AKT is activated via phosphorylation, it exerts regulatory control over GSK-3β [32]. In turn, GSK-3β can phosphorylate Nrf2, which facilitates Nrf2-Keap1 binding and drives Nrf2 toward ubiquitination and degradation, effectively suppressing its functional activity. Additionally, active GSK-3β alters the balance between Bax and Bcl-2. This shift impacts the permeability of the mitochondrial membrane, promoting cytochrome C release and subsequently triggering apoptotic cascades. Given that natural polysaccharides tend to possess complex chemical characteristics enabling multi-target regulation, the inhibitor did not completely block all beneficial effects, suggesting that the antioxidant and anti-fibrotic potential of PAP is partially mediated through this pathway, and other parallel potential crosstalk regulatory networks may also exist.

Despite these positive findings, among the tested fractions under the present experimental conditions, PAP exhibited the most pronounced protective activity against HG-induced cellular injury and DKD-associated renal damage. Nevertheless, the present study is limited in several respects that require further refinement in future work. First, although PAP has been chemically characterized by molecular weight and monosaccharide composition, further structural characterization, including glycosidic linkage analysis, NMR profiling, chromatographic fingerprinting, is still required to improve reproducibility and clarify the precise structure activity relationship. Second, the in vitro HG model design did not include an isotonic control, meaning that the 60 mM group does not represent pure glucotoxicity. Third, a single in vitro high-glucose environment cannot fully recapitulate the complex pathological microenvironment of type 2 diabetes in vivo, which includes advanced glycation end products (AGEs) and lipotoxicity. Moreover, due to current technical constraints, only the pharmacological blocker LY294002 was used, and direct genetic evidence based on specific gene knockout or knockdown is lacking. Finally, the precise transmembrane receptor species bound by PAP have not yet been identified through direct experiments. In the future, we will integrate multi-omics technologies to deeply elucidate the targeting network and receptor mechanisms by which PAP reverses DKD.

## 4. Materials and Methods

### 4.1. Chemicals and Reagents

*Phellodendron amurense* Rupr. was obtained from Yishunkang Medical Pharmacy (Jiamusi, China). Procell Life Science Technology Co., Ltd. (Wuhan, China) supplied the human kidney proximal tubular cell line (HK-2). All standards were obtained from Shanghai yuanye Bio-Technology Co., Ltd. (Shanghai, China). FBG and urinary protein kits were provided by Nanjing Jiancheng Bioengineering Institute. (Nanjing, China). H&E staining kit, Masson staining kit, PAS staining kit and TUNEL staining kit were purchased from Meilunbio (Dalian, China). Antibodies against HO-1, NQO1, Nrf2, p-AKT, p-GSK-3β, GSK-3β, α-SMA, collagen1, p-Smad2, Smad2, TGF-β, Bax and Bcl-2 were purchased from P Beyotime Biotechnology Co., Ltd. (Wuhan, China). All the other chemicals used were provided by Aladdin Chemical Reagent Co., Ltd. (Shanghai, China) and Beyotime Biotechnology Co., Ltd. (Wuhan, China).

### 4.2. Preparation and Characterization of Active Fractions

Dried *Phellodendron amurense* Rupr. slices weighing 0.5 kg were extracted under reflux three times for 2 h each with a solid-to-liquid ratio of 1:15 *w*/*v* using water, 0.5% HCl, or 60% ethanol, respectively. For polysaccharide preparation, the aqueous extract was concentrated and precipitated by adding ethanol to a final concentration of 85% *v*/*v* overnight. The resulting precipitate was deproteinized using papain followed by decolorization with AB-8 macroporous resin and lyophilization to yield refined PAP. Regarding total alkaloids, the acidic extract was neutralized with ammonia and adjusted to an ethanol content of 70% *v*/*v*. The supernatant was subsequently purified via D101 macroporous resin to obtain refined alkaloids. Finally, the 60% ethanolic extract was concentrated and purified using AB-8 macroporous resin followed by lyophilization to obtain the refined flavonoid fraction.

Total polysaccharide content was determined using the phenol-sulfuric acid method. Briefly, glucose standard solutions or the sample solution (0.1 mg/mL) were mixed with 5% phenol and concentrated sulfuric acid. After heating in a water bath for 15 min and cooling to room temperature, absorbance was measured at 490 nm. Total alkaloid content was assayed using the bromocresol green colorimetric method with berberine hydrochloride as the standard. The sample or standards were mixed with citrate-phosphate buffer (pH 4.0) and bromocresol green, followed by extraction with dichloromethane. The absorbance of the organic phase was measured at 417 nm after standing for 30 min. Total flavonoid content was evaluated using the NaNO2-Al(NO_3_)_3_-NaOH method with rutin as the standard. The sample solution or standards were sequentially reacted with 5% NaNO_2_, 10% Al(NO_3_)_3_, and 4% NaOH. After incubation for 20 min, absorbance was recorded at 510 nm. All contents were calculated based on their respective calibration curves.

### 4.3. Cell Culture

HK-2 cells were maintained in DMEM/F12 medium supplemented with 10% fetal bovine serum (FBS) and 1% penicillin-streptomycin in a humidified incubator at 37 °C with 5% CO_2_. For resuscitation, frozen cells were rapidly thawed in a 37 °C water bath, centrifuged at 1200 rpm for 5 min to remove the supernatant, resuspended in the complete medium, and seeded into 6 cm dishes. Upon reaching approximately 80% confluence, cells were washed twice with PBS and dissociated using trypsin at 37 °C. Digestion was terminated with complete medium when cells retracted, and the suspension was centrifuged (1200 rpm, 5 min) for subculturing. For cryopreservation, harvested cells were resuspended in freezing medium and stored at −80 °C following a gradient cooling process.

### 4.4. Construction of HG-Induced HK-2 Model and Screening of Effective Concentrations of Extracts

To establish the HG-induced model and screen the optimal concentrations of *Phellodendron amurense* Rupr. active fractions, the Cell Counting Kit-8 (CCK-8) assay was employed to evaluate cell viability. HK-2 cells were first exposed to varying concentrations of glucose (0–100 mM) for 24 or 48 h to determine the condition inducing approximately 50% viability inhibition. Subsequently, under the optimized HG conditions, cells were treated with total polysaccharides (0–400 µg/mL), total alkaloids (0–100 µg/mL), or total flavonoids (0–30 µg/mL) for 24 h. Following treatment, 10 µL of CCK-8 reagent was added to each well and incubated for 1 h. Absorbance was measured at 450 nm using a microplate reader, and the concentrations resulting in approximately 50% inhibition of cell viability were selected for subsequent experiments.

### 4.5. Measurement of Intracellular ROS in HK-2 Cells

Logarithmic growth phase HK-2 cells were rinsed with PBS (phosphate-buffered saline), digested with trypsin, and centrifuged to prepare a cell suspension. The cells were seeded in 6-well plates at a density of 5 × 10^5^ cells per well and cultured in an incubator for 24 h. Upon reaching approximately 70–90% confluence, the cells were randomly assigned to the following groups which were control group, HG group, HG + Polysaccharides group, HG + Alkaloids group, and HG + Flavonoids group. Cells in the treatment groups were co-incubated with HG and their respective active components at designated concentrations for 24 h. Following the treatments, the culture medium was discarded, and the cells were gently rinsed with PBS. Subsequently, 10 μmol/L 2′,7′-dichlorodihydrofluorescein diacetate was added as a fluorescent probe and incubated at 37 °C in the dark for 30 min. The plates were gently swirled every 5 min to ensure uniform interaction between the probe and the cells. The cells were washed twice with PBS to remove the residual extracellular probe. The intracellular ROS fluorescence was immediately observed and captured using an inverted fluorescence microscope, and the relative fluorescence intensity of each group was quantified using ImageJ software.

### 4.6. TUNEL Staining

The HK-2 cells were analyzed using the In Situ Cell Death Fluorescein Detection Kit via TUNEL assay. Images were captured under an optical microscope. The number of TUNEL-positive cells was quantified in each group of images. The percentage of positive cells was determined using ImageJ software version 1.54 (FIJI, National Institutes of Health, Bethesda, MD, USA).

### 4.7. Animals and Experimental Design

Fifty specific pathogen-free (SPF) male Sprague Dawley rats (6–8 weeks old, 200 ± 20 g) were obtained from the Animal Experimental Center of Jiamusi University. All animal procedures complied with the regulations of the Ethics Committee of Jiamusi University. Rats were housed under standard conditions (25 ± 2 °C, 50 ± 5% humidity, 12 h light and dark cycle) with ad libitum access to food and water. After one week of acclimatization, the rats were randomly divided into five groups (*n* = 10 per group): Control, DKD (2%STZ, 50 mg/kg), PAP (800 mg/kg), LY294002 group, and PAP + LY294002 groups. To induce the model, excluding the Control group, others group were fasted for 10 h and intraperitoneally injected with 2%STZ dissolved in 0.1 mol/L citrate buffer (pH 4.5, 4 °C). Diabetes was confirmed if fasting blood glucose (FBG) levels remained ≥16.7 mmol/L for three consecutive days after 72 h. These rats were subsequently fed a high-fat with high-sugar diet for 4 weeks. The DKD model was considered successfully established when rats exhibited sustained hyperglycemia and urine protein levels ≥ 30 mg. Following model establishment, rats were treated via oral gavage once daily for 8 weeks. The treatment groups received their respective drugs, while the control and DKD groups were administered an equivalent volume of vehicle (distilled water or saline). At the end of the experiment, rats were fasted for 12 h and anesthetized with 1% pentobarbital sodium (0.17 mL/100 g). Blood and fecal samples were collected, and rats were sacrificed by cervical dislocation. Kidney tissues were fixed in 4% paraformaldehyde, while other tissues were snap-frozen in liquid nitrogen and stored at −80 °C for further analysis.

### 4.8. Biochemical Analysis

FBG in serum and 24 h urinary protein levels were determined using specific commercial assay kits according to the manufacturer’s protocols. The final absorbance values for these assays were measured using a microplate reader.

### 4.9. Histological Examination

Kidney tissues were fixed in 4% paraformaldehyde, dehydrated in graded ethanol, and embedded in paraffin. 4–5 µm thick sections were prepared and stained with hematoxylin and eosin (HE), Masson’s trichrome, and Periodic Acid-Schiff (PAS) staining according to the manufacturer’s protocols. Histopathological changes were subsequently examined using a light microscope.

### 4.10. Immunofluorescence Analysis

Kidney tissue sections were routinely deparaffinized and dehydrated for transparency. After citrate-mediated antigen retrieval, sections were blocked with 5% BSA for 30 min and then incubated overnight at 4 °C with primary antibodies against Nrf2 (1:100) and p-GSK-3β (1:100). Following washing with PBS, FITC-conjugated (1:100) and Cy3-conjugated (1:100) secondary antibodies were applied and incubated for 1 h at 37 °C. Nuclei were counterstained with DAPI under dark conditions. Finally, a fluorescence microscope captured all images. The acquired images were analyzed for average optical density using ImageJ software.

### 4.11. Western Blot

Total protein was extracted from kidney tissues and HK-2 cells using radioimmunoprecipitation assay (RIPA) lysis buffer supplemented with 1 mM PMSF and protease/phosphatase inhibitor cocktails (Roche, Basel, Switzerland). To assess the nuclear translocation of Nrf2, nuclear and cytoplasmic fractions were isolated using a commercial Nuclear and Cytoplasmic Extraction Kit according to the manufacturer’s instructions. Protein concentration was quantified using a BCA Protein Assay Kit (Pierce Biotechnology, Rockford, IL, USA). Equal amounts of protein (30 μg) were separated by 10% or 12% SDS-PAGE and transferred onto polyvinylidene difluoride (PVDF) membranes. After blocking with 5% bovine serum albumin (BSA) in TBST for 2 h at room temperature, the membranes were incubated overnight at 4 °C with primary antibodies against PI3K, p-AKT, AKT, p-GSK-3β, GSK-3β, Nrf2, HO-1, NQO1, TGF-β, α-SMA, Collagen 1, Bcl-2, Bax, GAPDH, and LaminB. Subsequently, the membranes were incubated with HRP-conjugated secondary antibodies for 1.5 h at room temperature. Protein bands were visualized using an enhanced chemiluminescence (ECL) kit and captured by an imaging system. Band density was quantified using ImageJ software. Target protein levels were normalized to GAPDH or LaminB, while phosphorylated proteins were normalized to their respective total protein levels.

### 4.12. Statistical Analysis

GraphPad Prism 9.0 (GraphPad Software Inc., San Diego, CA, USA) was used for data analysis and plotting. Parametric comparisons between study groups were conducted using one-way analysis of variance (ANOVA) followed by Tukey’s post hoc test for multiple comparisons. Data were expressed as mean ± standard deviation (SD), with *p* < 0.05 indicating statistically significant differences.

## 5. Conclusions

In summary, this study demonstrates that the polysaccharides and flavonoids derived from *Phellodendron amurense* Rupr. exhibited protective effects in alleviating DKD, Among the tested fractions under the present experimental conditions, PAP exhibited the most pronounced protective activity. PAP ameliorates DKD symptoms by enhancing antioxidant capacity, mitigating renal fibrosis, and reducing cellular apoptosis, with these protective effects being partially dependent on the activation of the PI3K/AKT/GSK-3β/Nrf2 pathway.

## Data Availability

The original contributions presented in this study are included in the article. Further inquiries can be directed to the corresponding authors.

## Supplementary Materials

The following supporting information can be downloaded at: https://www.mdpi.com/article/10.3390/ph19060965/s1, Figure S1: Original uncropped Western blot images corresponding to Figure 3 and Figure 6.

## Abbreviations

The following abbreviations are used in this manuscript:

PAP	*Phellodendron amurense* Rupr. polysaccharides
DKD	Diabetic kidney disease
HG	High-glucose
ROS	Reactive oxygen species
TUNEL	TdT-mediated dUTP Nick-End Labeling
WB	Western Blot
IDF	International Diabetes Federation
ECM	Renal extracellular matrix
SPF	Specific pathogen-free
FBG	Fasting blood glucose
PAS	Periodic Acid-Schiff
HE	Hematoxylin and eosin
RIPA	Radioimmunoprecipitation assay
PVDF	Polyvinylidene difluoride
