# Supplementary material for: Comparative Study of Phellodendron amurense Rupr. Components in Alleviating Diabetic Kidney Disease via the PI3K/AKT/GSK-3β/Nrf2 Pathway
Source: Pharmaceuticals (Basel). 2026 Jun 22;19(6):965. doi: 10.3390/ph19060965 (PMC13305558; doi:10.3390/ph19060965)
Supplement: Supplementary file 1 [file pharmaceuticals-19-00965-s001.zip › pharmaceuticals-4271710-supplementary.pdf]

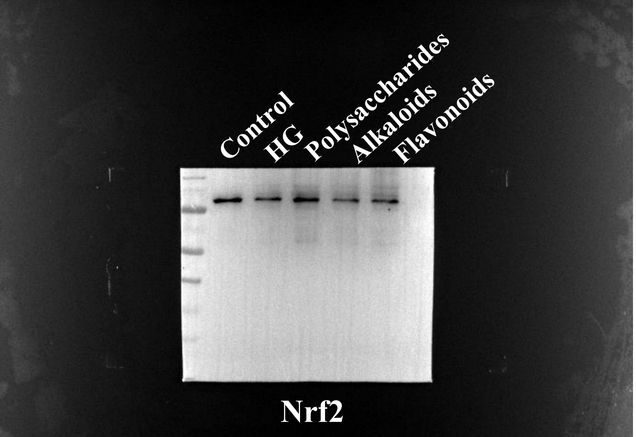

Figure 3.A

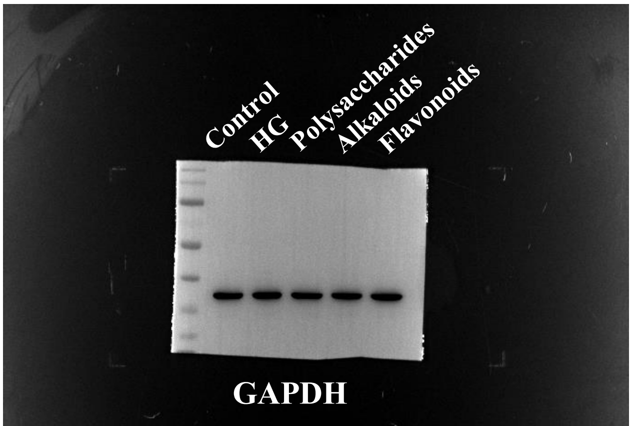

Figure 3.A

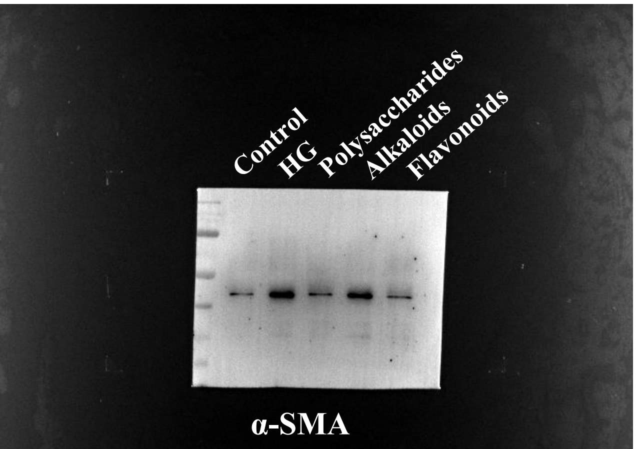

Figure 3.B

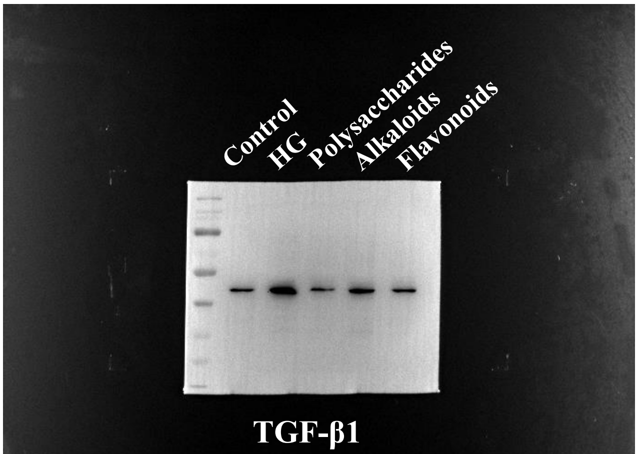

Figure 3.B

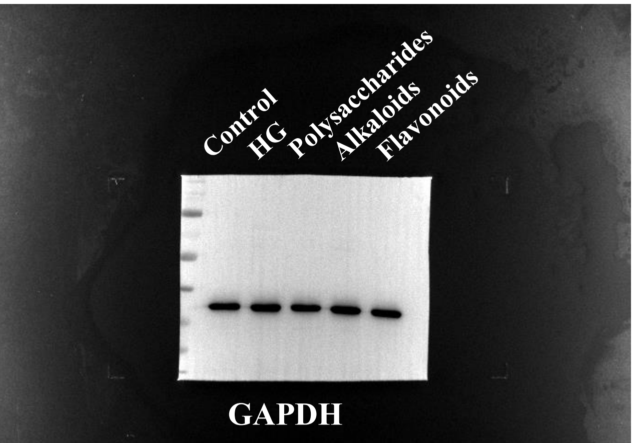

Figure 3.B

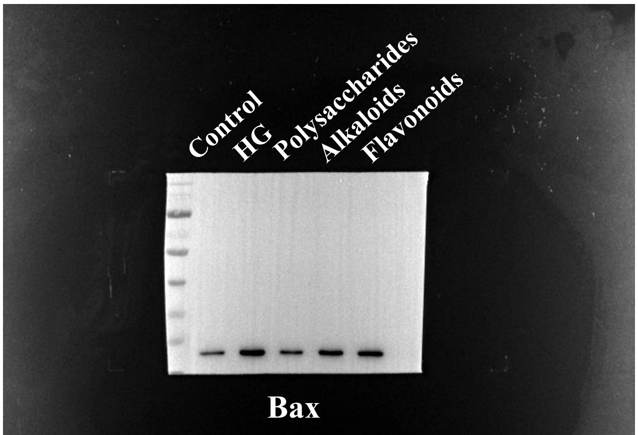

Figure 3.C

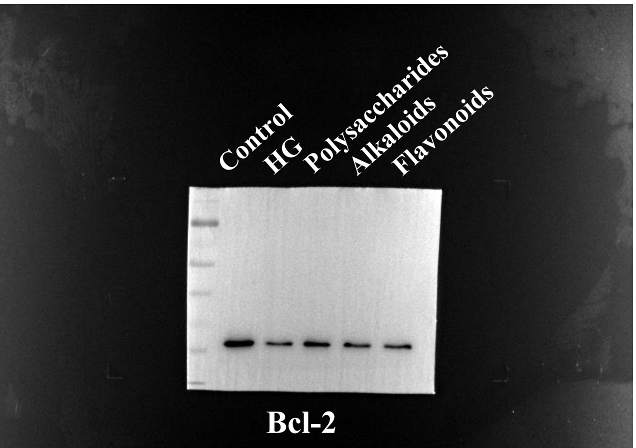

Figure 3.C

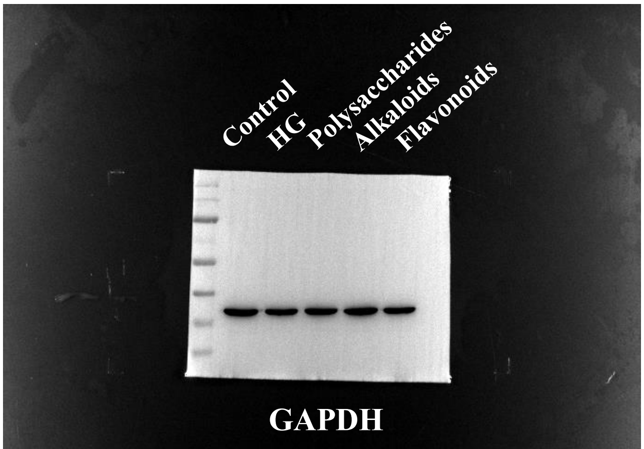

Figure 3.C

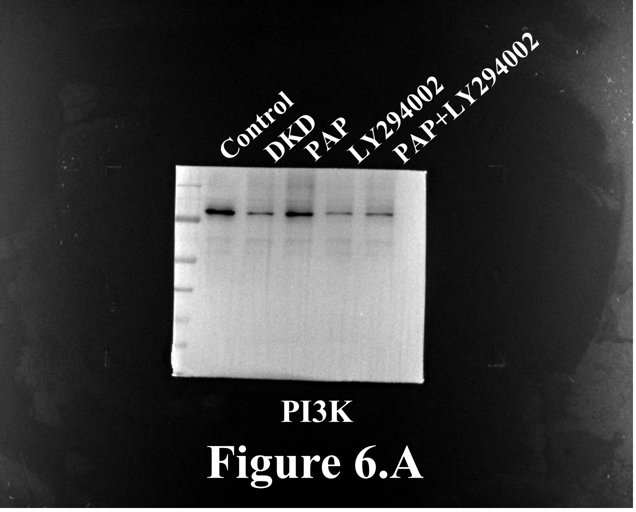

Figure 6.A

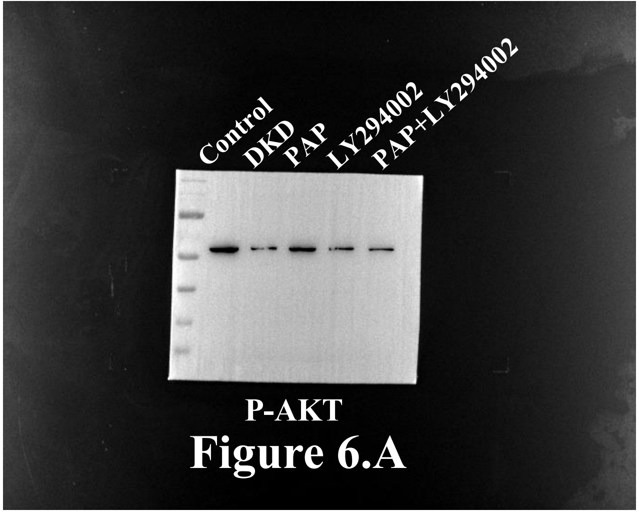

Figure 6.A

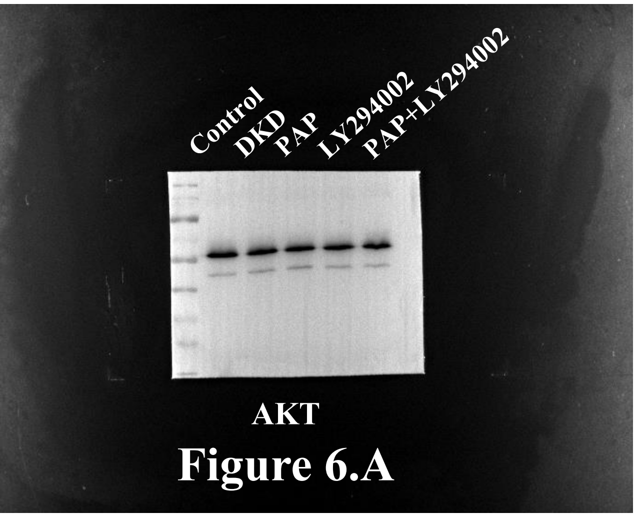

Figure 6.A

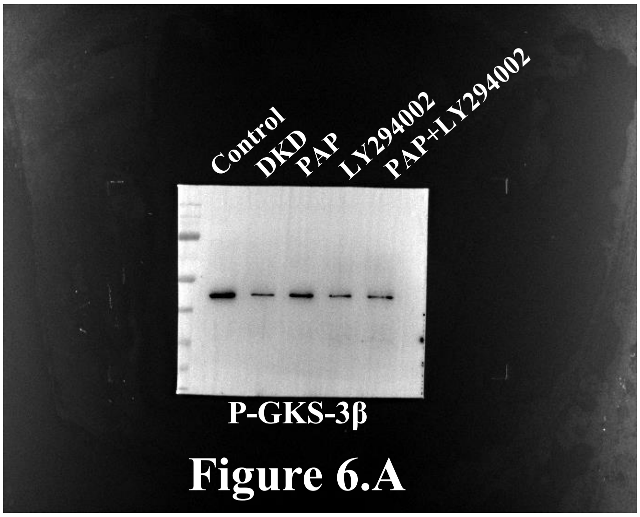

Figure 6.A

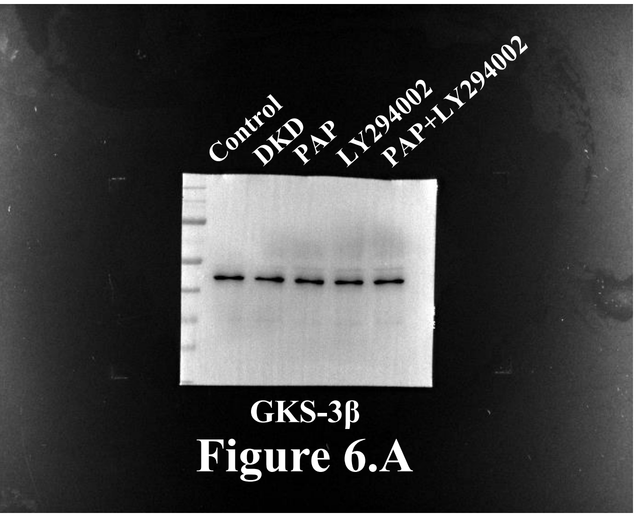

Figure 6.A

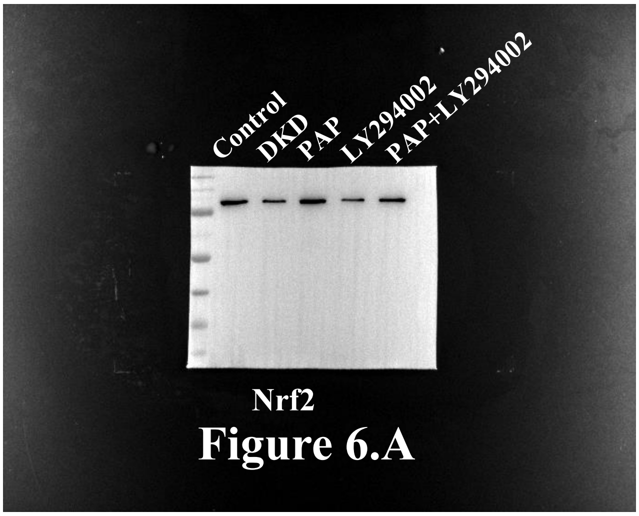

Figure 6.A

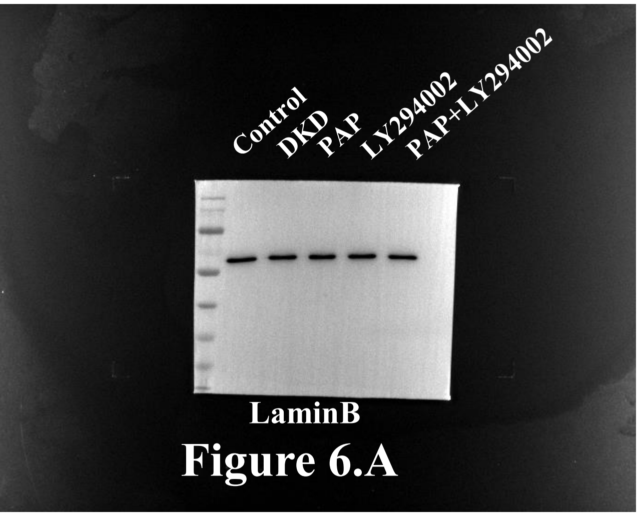

Figure 6.A

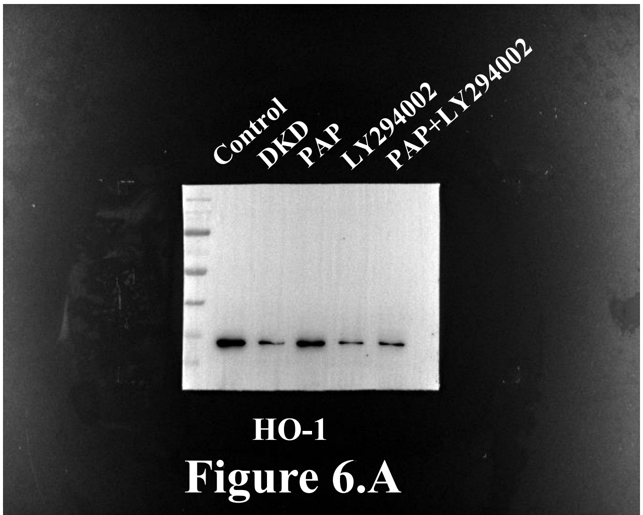

Figure 6.A

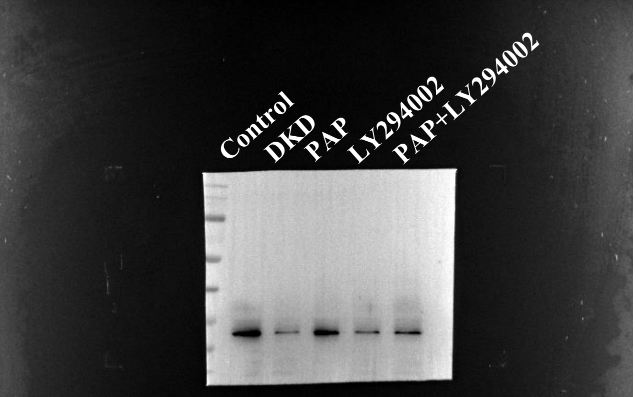

NOQ1

Figure 6.A

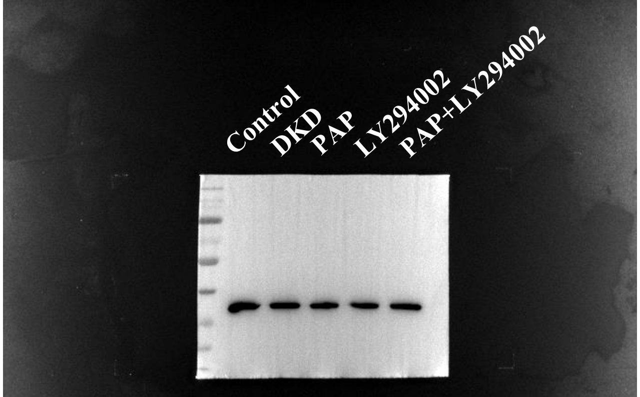

GAPHD

Figure 6.A

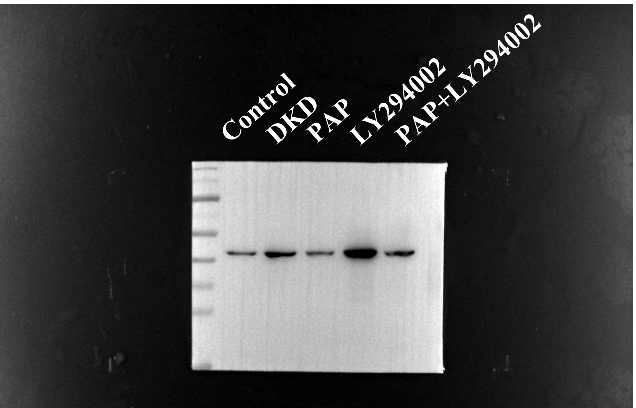

α-SMA

Figure 6.B

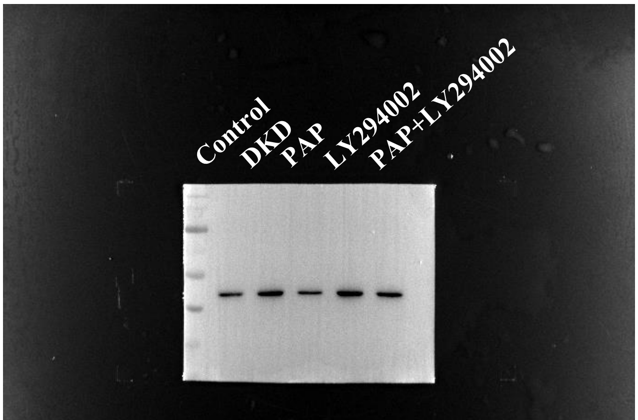

TGF-β1

Figure 6.B

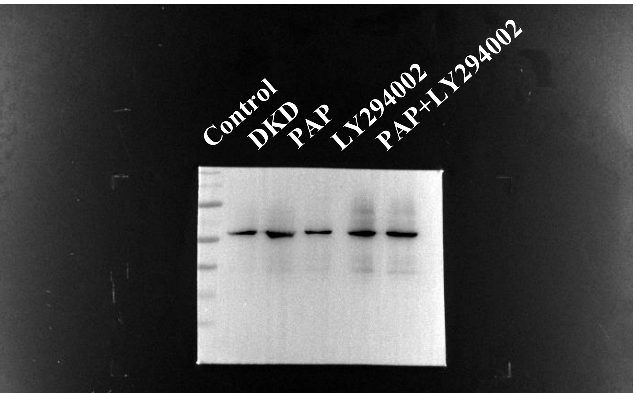

P-SMAD2

Figure 6.B

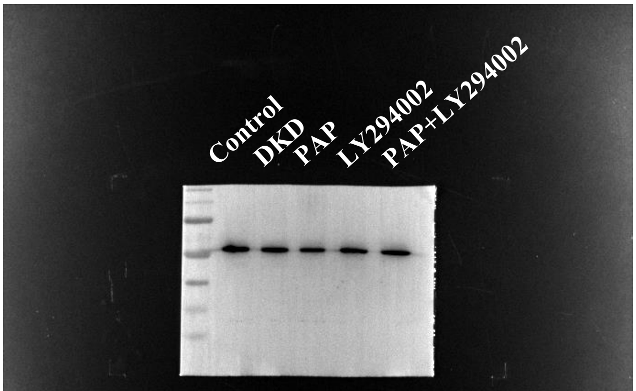

SMAD2

Figure 6.B

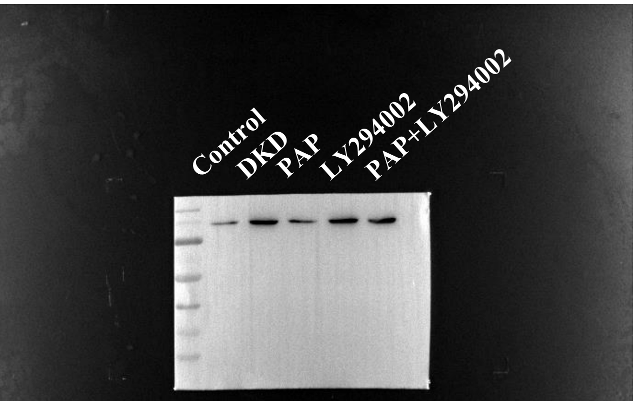

Collagen 1

Figure 6.B

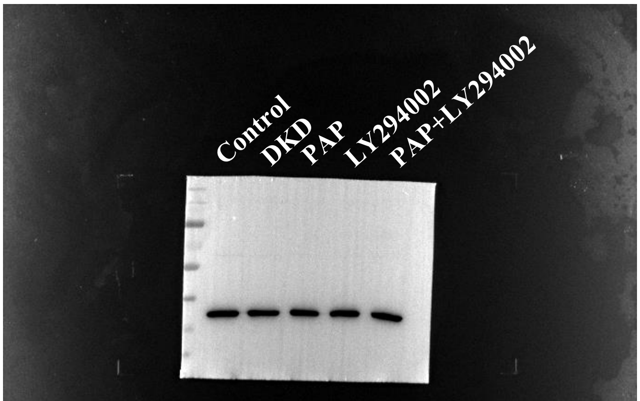

GAPHD

Figure 6.B

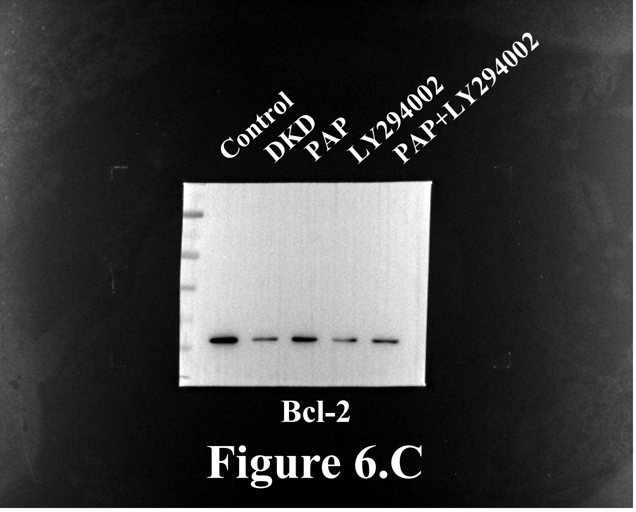

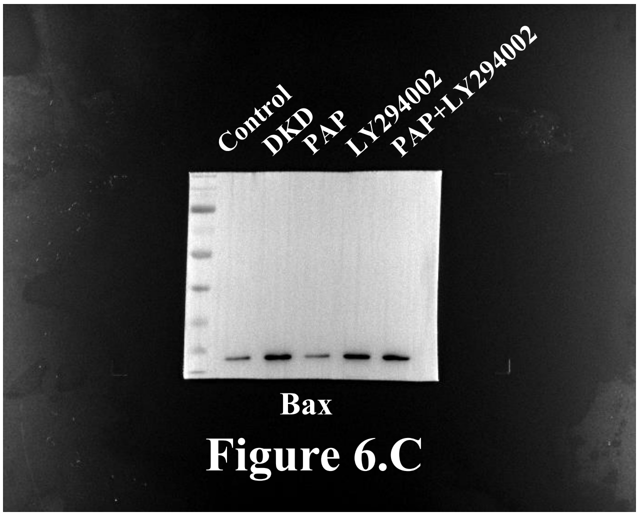

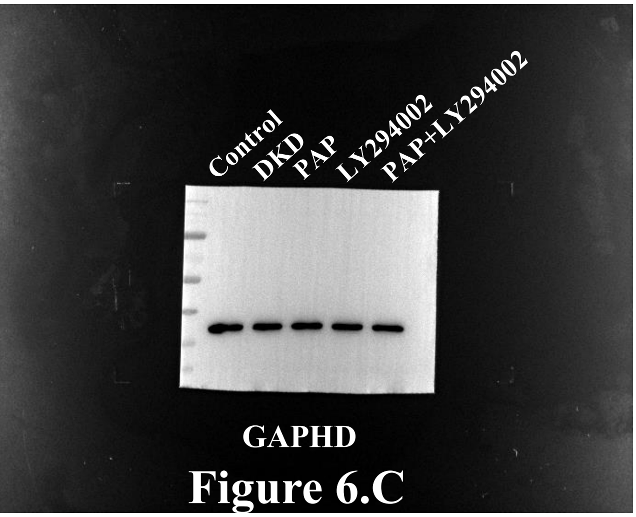

Figure S1. Original uncropped Western blot images corresponding to Figure 3 and Figure 6.
